# Computed Tomography-Based Radiomics Nomogram: Potential to Predict Local Recurrence of Gastric Cancer After Radical Resection

**DOI:** 10.3389/fonc.2021.638362

**Published:** 2021-09-02

**Authors:** Liebin Huang, Bao Feng, Yueyue Li, Yu Liu, Yehang Chen, Qinxian Chen, Changlin Li, Wensi Huang, Huimin Xue, Xuehua Li, Tao Zhou, Ronggang Li, Wansheng Long, Shi-Ting Feng

**Affiliations:** ^1^Department of Radiology, Jiangmen Central Hospital, Jiangmen, China; ^2^School of Electronic Information and Automation, Guilin University of Aerospace Technology, Guilin, China; ^3^Department of Radiology, The First Affiliated Hospital of Jinan University, Guangzhou, China; ^4^Department of Radiology, The First Affiliated Hospital of Sun Yat-sen University, Guangzhou, China; ^5^Department of Pathology, Jiangmen Central Hospital, Jiangmen, China

**Keywords:** gastric cancer, computed tomography, radiomics signature, local recurrence (LR), nomogram

## Abstract

**Objective:**

Accurate prediction of postoperative recurrence risk of gastric cancer (GC) is critical for individualized precision therapy. We aimed to investigate whether a computed tomography (CT)-based radiomics nomogram can be used as a tool for predicting the local recurrence (LR) of GC after radical resection.

**Materials and Methods:**

342 patients (194 in the training cohort, 78 in the internal validation cohort, and 70 in the external validation cohort) with pathologically proven GC from two centers were included. Radiomics features were extracted from the preoperative CT imaging. The clinical model, radiomics signature, and radiomics nomogram, which incorporated the radiomics signature and independent clinical risk factors, were developed and verified. Furthermore, the performance of these three models was assessed by using the area under the curve (AUC) of receiver operating characteristic (ROC) curve analysis and decision curve analysis (DCA).

**Results:**

The radiomics signature, which was comprised of two selected radiomics features, namely, contrast_GLCM and dissimilarity_GLCM, showed better performance than the clinical model in predicting the LR of GC, with AUC values of 0.83 in the training cohort, 0.84 in the internal validation cohort, and 0.73 in the external cohort, respectively. By integrating the independent clinical risk factors (N stage, bile acid duodenogastric reflux and nodular or irregular outer layer of the gastric wall) into the radiomics signature, the radiomics nomogram achieved the highest accuracy in predicting LR, with AUC values of 0.89, 0.89 and 0.80 in the three cohorts, respectively. DCA in the validation cohort showed that radiomics nomogram added more net benefit than the clinical model within the range of 0.01-0.98.

**Conclusion:**

The CT-based radiomics nomogram has the potential to predict the LR of GC after radical resection.

## Introduction

Gastric cancer (GC) remains one of the leading causes of cancer-related deaths globally, especially in Eastern Asia (particularly in Korea, Mongolia, Japan, and China) (1). Surgical resection continues to remain the best curative treatment for GC. With advances in surgical techniques and equipment along with neoadjuvant therapies, even advanced-stage cases have become amenable to surgical resection. However, recurrence after curative gastrectomy, continues to severely affect the long-term outcomes, with a reported incidence as high as 36.9% to 45.9% (2, 3). The proportion of local recurrence (LR) cases in all the recurrent cases can be as high as 53.7% (4). Once relapse occurs, the patients have very few treatment options. Thus, how to decrease the incidence of LR is a significant clinical problem. According to two randomized phase III trials in Korea and Japan, postoperative adjuvant chemotherapy showed a survival benefit for the patients with locally advanced GC following D2 gastrectomy while comparing with those surgery alone, and it can also decrease the incidence of recurrence (5, 6). Another intergroup trial demonstrated that postgastrectomy chemoradiation can significantly reduce the high LR rate of GC, suggesting that all patients at high risk for recurrence should accept postgastrectomy chemoradiation (7). How to identify these patients is a crucial problem. Thus, it is necessary to develop a reliable prediction tool to identify patients at high risk of LR of GC after radical resection.

The tumor, lymph node, metastasis (TNM) staging system is widely used for risk stratification and treatment plan-making of GC patients. The patients, with stage II or higher stage GC, were recommended to accept postoperative chemotherapy (6). However, the clinical outcomes often vary, even in patients with the same TNM stage. Administration of postoperative chemotherapy to all these patients is unnecessary and may even be harmful for some patients (8). Previous study have reported that the TNM staging system lacks the ability to include various other tumor and patient characteristics, which are required to enable individualized predictions (8).

In recent years, radiomics has attracted increasing attention by researchers, this method can extract sub-visual yet quantitative features from radiological images by using many indices, including intensity as well as texture and shape features, to reflect biological information such as cell morphology, gene and molecular expression, and tumor heterogeneity (9). Currently, radiomics is widely used in cancer diagnosis; prediction of therapeutic response and recurrence; and prognostication of survival, especially in lung carcinoma, colorectal carcinoma, and breast cancer (10–12). However, few studies have focused on the application of radiomics in GC, of the available studies, most of them have focused on the use of radiomics for predicting the therapeutic response and survival in GC (13, 14). Jiang et al. reported that radiomics signature of computed tomography imaging is a powerful predictor of overall survival and disease-free survival (14). Another study by Li et al. used a single center data to evaluate the prognostic value of computed tomography radiomics features in patients with GC following curative resection, the results showed that the radiomics features were useful in stratifying patients with GC following radical resection into high- and low-risk groups (13). However, to our knowledge, none of these studies had focused on evaluating the risk of LR.

The current study, using two center data, was conducted to investigate whether a computed tomography (CT)-based radiomics nomogram, which combined the radiomics signature and clinical risk factors, can be used as a tool for predicting the LR of GC after radical resection.

## Materials and Methods

### Patients

A total of 272 consecutive patients in center 1, who had underwent gastrectomy and pathologically proven GC from October 2008 to July 2017, were enrolled in this study. In addition, we also collected an external validation cohort from center 2, including 70 patients from March 2015 to April 2017. The enrollment procedure is shown in the **Supplementary Material Figure S1**. The inclusion and exclusion criteria of the two centers were the same. The inclusion criteria were as follows: (a) the patients who had received gastrectomy, (b) the patients had pathologically proven GC, (c) the patients had preoperative abdominal contrast-enhanced CT examination. The exclusion criteria were as follows: (a) the contrast-enhanced abdominal CT examination longer than two weeks before operation, (b) the patients had unsatisfactory gastric distention and insufficient quality of CT imaging, (c) the surgery didn’t meet the standard of D2 lymphadenectomy and R0 resection, (d) less than 15 retrieved lymph nodes, (e) incomplete medical records, (f) had previous treatment with chemotherapy or radiotherapy before surgery, (g) follow-up shorter than 2 years before LR. This study was reviewed and approved by the institutional review board of Jiangmen central hospital and the First Affiliated Hospital of Sun Yat-sen University. Due to the retrospective nature, the ethics committees waived the requirement of written informed consent for participation.

We further divided these 272 patients from center 1 into training cohort and internal validation cohort. The training cohort consisted of 194 patients enrolled from October 2008 to August 2015, while the validation cohort consisted of 78 patients enrolled from September 2015 to July 2017. The clinicopathological data of each patient, including sex, age, tumor location, bile acid duodenogastric reflux after radical resection, histological classification, histological grade, depth of tumor involvement (T stage), involvement of regional lymph nodes (N stage), TNM stage, lymphovascular invasion, lauren classification, borrmann type and postoperative chemotherapy were derived from the electronic medical records. The CT imaging data of the patients were acquired from the database of two centers *via* picture archiving and the communication system.

### CT Acquisition

Abdominal contrast-enhanced CT was performed using one of the following CT scanners: Toshiba Aquilion One-64 (Toshiba Medical Systems, Otawara, Japan) and Somatom Force CT Scanner (Siemens Healthcare). The scanning parameters were as follows: tube voltage, 120 kv; tube current, auto; detector collimation, 64 × 0.625 mm and 192 × 0.625 mm, respectively; field of view, 350 mm × 350 mm; pitch, 0.656 and 0.7, respectively; rotation time, 0.5 s; matrix, 512 × 512; slice interval, 3 mm; slice thickness, 3 mm; reconstructed section thicknesses, 3 mm. Triple-phase CT, which included a plain scan, arterial phase, and portal venous phase, was performed for each patient. The arterial and portal venous phase images were acquired at 30 s and 60 s, respectively, after the injection of contrast agent (1.5 mL/kg, 3.0–3.5 mL/s, Ultravist, Bayer Schering Pharma, Berlin, Germany) *via* a pump injector.

### CT image evaluation

The CT images of the patients were evaluated by two radiologists (reader 1 and reader 2, with 10 years and 15 years of experience in abdominal imaging diagnosis, respectively), who were only aware of the diagnosis and location of GC. They first independently evaluated the transverse, coronal and sagittal CT images of patients on the PACS system. In the evaluation process, different CT window width and window level was adjusted to better display each CT sign, and the corresponding CT signs appear in any position were considered positive. Finally, the evaluation results of the two doctors were summarized, and the cases with different opinions were reviewed and discussed together to resolve differences.

The following CT signs were evaluated: (a) high enhanced serosa sign (present or absent), high enhancement with long ribbon shape or patchy shape on the side of gastric serous around the lesion. (b) nodular or irregular outer layer of the gastric wall (present or absent), nodular or irregular outer layer on the side of gastric serous around the lesion. (c) perigastric fat infiltration (present or absent), increased density of the fatty layer around the lesion, with or without linear and reticular structure. (d) tumor necrosis (present or absent), non-enhanced areas within the lesion. (e) perigastric lymph node necrosis (present or absent), non-enhanced areas within the perigastric lymph node.

### Pathological Evaluation

All of the surgical specimens were examined by a senior pathologist (with 16 years of experience in gastrointestinal pathology) and reclassified as per the American Joint Committee on Cancer 8th edition TNM staging system (2016) (15). The histological classification, histological grade, T stage, N stage, TNM stage, lymphovascular invasion, and Lauren classification were assessed.

### Definition and Surveillance of Bile Acid Duodenogastric Reflux

Bile acid duodenogastric reflux was defined as the reverse flow of bile from the duodenum into the remnant stomach. In the present study, the bile acid duodenogastric reflux was monitored by endoscopy. The patient was considered to have bile acid duodenogastric reflux if the following conditions were satisfied: (a) the presence of yellow or yellowish-green bile on the surface of the gastric mucosa or mixed in the gastric juice without obvious vomiting symptoms during the examination, (b) visualization of bile reflux through the afferent limb into the remnant stomach during the gastroscopy for more than 1 min.

### Follow-up and definition of LR

LR was defined as reappearance of cancer at the gastrojejunostomy site, tumor bed, residual stomach, duodenal stump, and/or regional lymph nodes (16). Recurrence in the gastrojejunostomy site, tumor bed, residual stomach and duodenal stump was pathologically confirmed by endoscopic biopsy. The regions of lymph node recurrence were determined according to the Japanese Gastric Cancer Treatment Guidelines (17).

The endpoint of this study was LR. According to the National Comprehensive Cancer Network Clinical Practice Guidelines in Oncology, Gastric Cancer Version 2.2019 (18), all patients included in this study were followed up for at least two years if LR didn’t occur. The patients were followed up in the outpatient department of our hospitals every 3–6 months in the first 2 years, every 6–12 months during the next 3 years, and then yearly. Abdominal contrast-enhanced CT, endoscopy, and tumor biomarker tests were performed in order to detect postoperative recurrence. The tumor biomarker tests were performed at every follow-up visit. A CT examination was performed every 6–12 months for the first 2 years, and then yearly. Endoscopy was performed if LR was suspected on CT or based on the symptoms of the patient. Endoscopic biopsy and pathological examination were performed if the patient was suspected of having LR on endoscopy.

### Tumor Segmentation and Extraction of Radiomics Features

Tumor segmentation was performed in the portal venous phase of preoperative CT imaging. The region of interest (ROI) was manually segmented by a professional radiologist (reader 1) using our in-house software developed with MATLAB 2016 (Mathworks, Natick, MA, USA). Contour lines of the ROI were drawn along the boundaries of the tumor on each axial image, avoiding the adjacent air and fat. Subsequently, reconstruction of the whole tumor volume was performed using the identified axial images. In order to enhance the robustness and repeatability of extraction of the radiomics features, wavelet bandpass filtering, isotropic resampling, and grayscale discretization were applied while reconstructing the whole tumor volume. Features were extracted using the in-house software developed with MATLAB 2016 (Mathworks, Natick, MA, USA). The extracted radiomics features were normalized by the z-score (19).

Intraclass correlation coefficients (ICCs) were used to evaluate the reproducibility and stability of the radiomics parameters. Reader 1 performed the segmentation on all images of the patients. Reader 2 randomly chose 30 patients from the training cohort and performed tumor segmentation for inter-reader agreement analysis. The radiomics parameters with ICC values greater than 0.75 were considered to be reliable.

### Selection of Feature Parameters and Building of the Radiomics Signature

The least absolute shrinkage and selection operator (LASSO) logistic regression was used to select the features, and the features with non-zero coefficients were considered as valuable predictors for predicting the LR of GC. The process of feature selection based on the LASSO was showed in **Supplementary Material A1**. Subsequently, the non-zero coefficients of the selected features were used to build a radiomics signature by using the linear combination (**Supplementary Material Table S1**). The output of the radiomics signature was labeled as the radiomics score (R-score). Differences of the radiomics signature between the LR group (RG) and the nonrecurrence group (NRG) were assessed by the Mann–Whitney U test.

### Construction of the Clinical Model

Univariate analysis was performed using the various clinicopathological parameters to identify the significant factors associated with the LR of GC. Subsequently, multivariate logistic regression analysis was used to build the clinical model.

### Construction of the Radiomics Nomogram

A combined model, which integrated the radiomics signature and the clinical risk factors, was constructed using multivariate logistic regression analysis and finally presented as a radiomics nomogram. Backward step-wise selection, accompanied by the likelihood ratio test as the stopping rule, was performed while constructing the combined model. Calibration of the nomogram was acquired from the calibration curve analysis, and the goodness of fit was calculated using the Hosmer–Lemeshow test.

### Predictive Performance Evaluation of Each Model

Receiver operating characteristic (ROC) curve analysis was performed to evaluate the performance of each model. The area under the curve (AUC), sensitivity, specificity, accuracy, positive predictive value (PPV), negative predictive value (NPV), and optimal threshold for each model were recorded. A larger AUC represented a higher prediction accuracy. Comparison of the AUCs of the prediction models was accomplished by the DeLong test.

The net reclassification index (NRI) was used to assess the performance between the clinical model and the radiomics nomogram. Moreover, stratified analysis was performed on the patients’ age, sex, CT system, and contrast agent.

### Clinical Utility Analysis of the Prediction Models

Decision curve analysis (DCA) was used to evaluate the clinical utility of the prediction models by quantifying the net benefits at different threshold probabilities (20).

### Statistical Analysis

The Mann–Whitney U test was used to compare the continuous variables such as age and R-scores between the RG and NRG in the training and validation cohorts. In addition, the chi-squared test was used to compare the categorical data such as sex, tumor location, incidence of bile acid duodenogastric reflux, histological classification, histological grade, T stage, N stage, TNM stage, lymphovascular invasion, Lauren classification, Borrmann type and postoperative chemotherapy.

All statistical analyses were performed using R3.0.1 (http://www.rproject.org) and MATLA2016. The “glmnet”, “pROC”, and “dca.r.” packages were used to accomplish the LASSO logistic regression analysis, ROC curve analysis, and DCA, respectively. The differences were considered to be statistically significant if the P value was less than 0.05.

## Results

### Patients and Clinical Data

The median follow-up time was 43.5 months (range 6 to 72 months). The clinical data of the patients of three cohorts are presented in **Table 1**. The sex, age, tumor location, type of tumor differentiation, and incidence of lymphovascular invasion were similar in the RG and NRG in the training cohorts (**Table 1**). However, the incidence of bile acid duodenogastric reflux, histological classification, T stage, N stage, TNM stage, Lauren classification, Borrmann type, postoperative chemotherapy, high enhanced serosa sign and nodular or irregular outer layer of the gastric wall were significantly different between the RG and NRG, in all the three cohorts (P < 0.05 for all these clinical factors) (**Table 1**).

**Table 1:** Clinicopathological characteristics of the RG and the NRG in the training and validation cohorts.

Characteristic	Training cohort (n = 194)			Internal validation cohort (n = 78)			External validation cohort (n=70)		
	RG (n = 37)	NRG (n = 157)	P value	RG (n = 19)	NRG (n = 59)	P value	RG (n = 9)	NRG (n = 61)	P value
**Sex**									
Male	22	100	0.631	10	35	0.608	6	36	0.662
Female	15	57		9	24		3	25	
**Age, years** (mean ± SD)	58.51 ± 11.85	60.38 ± 11.37	0.553	60.79 ± 10.91	60.00 ± 13.17	0.616	60.56 ± 12.21	59.57 ± 13.81	0.841
**Tumor location**									
Upper 1/3	5	20	0.990	8	8	0.058	3	14	0.161
Middle 1/3	9	39		3	11		1	14	
Lower 1/3	22	95		8	39		2	27	
Multiple	1	3		0	1		3	6	
**Bile acid duodenogastric reflux**									
Present	22	30	<0.001*	8	9	0.032*	6	7	<0.001*
Absent	15	127		11	50		3	54	
**Histological classification**									
Poorly differentiated adenocarcinoma	20	61	0.028*	1	22	0.036*	2	33	<0.001*
Tubular adenocarcinoma	6	46		8	22		5	20	
Mucinous adenocarcinoma	0	16		5	5		1	1	
Papillary adenocarcinoma	0	5		2	1		0	1	
Signet-ring cell carcinoma	11	25		3	8		1	6	
Others	0	4		0	1		0	0	
**Histological grade**									
G1	0	7	0.159	0	3	0.190	0	1	0.290*
G2	5	36		9	16		5	18	
G3	32	114		10	40		4	42	
**T stage**									
T1a	0	4	0.002*	0	3	0.047*	0	0	0.014*
T1b	0	9		0	0		0	0	
T2	1	19		0	2		0	17	
T3	29	116		10	11		6	14	
T4a	6	3		8	41		3	30	
T4b	1	6		1	2		0	0	
**N stage**									
N0	4	53	<0.001*	4	16	0.047*	1	25	0.019*
N1	3	30		0	6		0	6	
N2	12	26		6	13		7	14	
N3a	9	38		2	17		1	9	
N3b	9	10		7	7		0	7	
**TNM stage**									
IA	0	11	0.004*	0	2	0.014*	0	0	0.043*
IB	0	8		0	0		0	13	
IIA	4	40		3	2		0	5	
IIB	3	24		1	15		2	14	
IIIA	11	29		6	16		2	13	
IIIB	10	34		4	21		5	8	
IIIC	9	11		5	3		0	8	
**Lymphovascular invasion**									
Present	10	52	0.475	9	19	0.231	5	13	0.028*
Absent	27	105		10	40		4	48	
**Lauren classification**									
Intestinal type	3	42	0.041*	11	16	0.003*	1	26	0.020*
Mixed type	8	34		3	10		0	11	
Diffuse type	26	81		5	33		8	24	
**Borrmann type**									
I	4	1	0.008*	3	2	0.122	1	1	0.025*
II	3	27		1	6		1	2	
III	24	111		14	39		6	58	
IV	6	18		1	12		1	0	
**Postoperative chemotherapy**									
Present	29	89	0.025*	8	37	0.011*	4	50	0.038*
Absent	8	68		11	22		5	11	
**High enhanced serosa sign**									
Present	27	86	0.043*	10	45	0.049*	8	33	0.048*
Absent	10	71		9	14		1	28	
**Nodular or irregular outer layer of the gastric wall**									
Present	28	87	0.024*	10	46	0.033*	8	32	0.039*
Absent	9	70		9	13		1	29	
**Perigastric fat infiltration**									
Present	22	78	0.063	12	35	0.766	6	28	0.402
Absent	15	79		7	24		3	33	
**Tumor necrosis**									
Present	7	19	0.274	6	4	0.005*	0	2	0.582
Absent	30	138		13	55		9	59	
**Perigastric lymph node necrosis**									
Present	10	15	0.004*	7	9	0.043*	0	2	0.582
Absent	27	142		12	50		9	59	
**Radiomics score: median (interquartile range)**	-0.86 (-1.32 to -0.32)	-1.77 (-2.21 to -1.29)	<0.001*	-1.37 (-1.57 to -0.91)	-1.96 (-2.31 to -1.68)	<0.001*	-1.41 (-1.45 to -1.40)	-1.45 (-1.45 to -1.44)	0.001*

RG, recurrence group. NRG, nonrecurrence group. The differences in age and radiomics score were assessed by the Mann–Whitney U-test. The differences of sex, tumor location, bile acid duodenogastric reflux, histological classification, histological grade, T stage, N stage, TNM stage, Lauren classification, Borrmann type, postoperative chemotherapy, high enhanced serosa sign, nodular or irregular outer layer of the gastric wall, perigastric fat infiltration, tumor necrosis, and perigastric lymph node necrosis were assessed by the chi-squared test. SD, standard deviation. *Statistically significant. The radiomics score is presented as the interquartile range.

### Selection of Radiomics Features and Building of the Radiomics Signature

**Figure 1:**
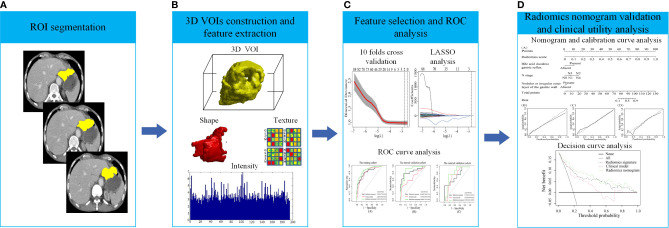
The process of data analysis. **(A)** Region of interest (ROI) segmentation of gastric cancer (GC) lesions. **(B)** Three-dimensional reconstruction of the segmented GC lesions and feature extraction. **(C)** Feature selection and performance of the receiver operating characteristic (ROC) curve. **(D)** Performance of the radiomics nomogram and clinical utility.

**Figure 1** shows the process of data analysis and selection of radiomics features. A total of 10,324 three-dimensional features, including the shape, intensity and texture of the tumor on CT, were extracted in the current study. Subsequently, a total of 734 recurrence-related features among the above features set, which was statistically different between the RG and NRG in the training cohort, with ICC values >0.75 (ICC = 0.75–0.99), were selected for further LASSO logistic regression analysis. Finally, two recurrence-related features, namely, contrast gray level co-occurrence matrix (GLCM) and dissimilarity_GLCM, were selected as valuable predictors to build the radiomics score calculation formula (**Table 2** and **Figure 1C**). The radiomics score calculation formula and the selected recurrence-related features are presented in **Supplementary Material Table S1**. The radiomics score showed a statistically significant difference between the RG and NRG in both the training and validation cohorts (P < 0.001).

**Table 2:** Radiomic features of the RG and the NRG in the training and validation cohorts.

Radiomic features	Training cohort (n = 194)			Internal Validation Cohort (n = 78)			External validation cohort (n = 70)		
	RG (n = 37)	NRG (n = 157)	P value	RG (n = 19)	NRG (n = 59)	P value	RG (n = 9)	NRG (n = 61)	P value
Contrast_GLCM_	21.93 ± 6.79	27.15 ± 11.28	0.001*	21.55 ± 4.34	27.23 ± 9.11	<0.001*	33.86 ± 10.62	30.09 ± 11.72	0.003*
1_1.2_Lloyd_32									
Dissimilarity_GLCM_	3.25 ± 0.59	3.64 ± 0.84	0.002*	3.26 ± 0.41	3.74 ± 0.57	<0.001*	4.32 ± 0.74	4.01 ± 0.85	0.002*
1_1.2_Lloyd_32									

Data are presented as the mean ± standard deviation. The P value is derived by the Mann-Whitney U test. *P < 0.05.

**Figure 2:**
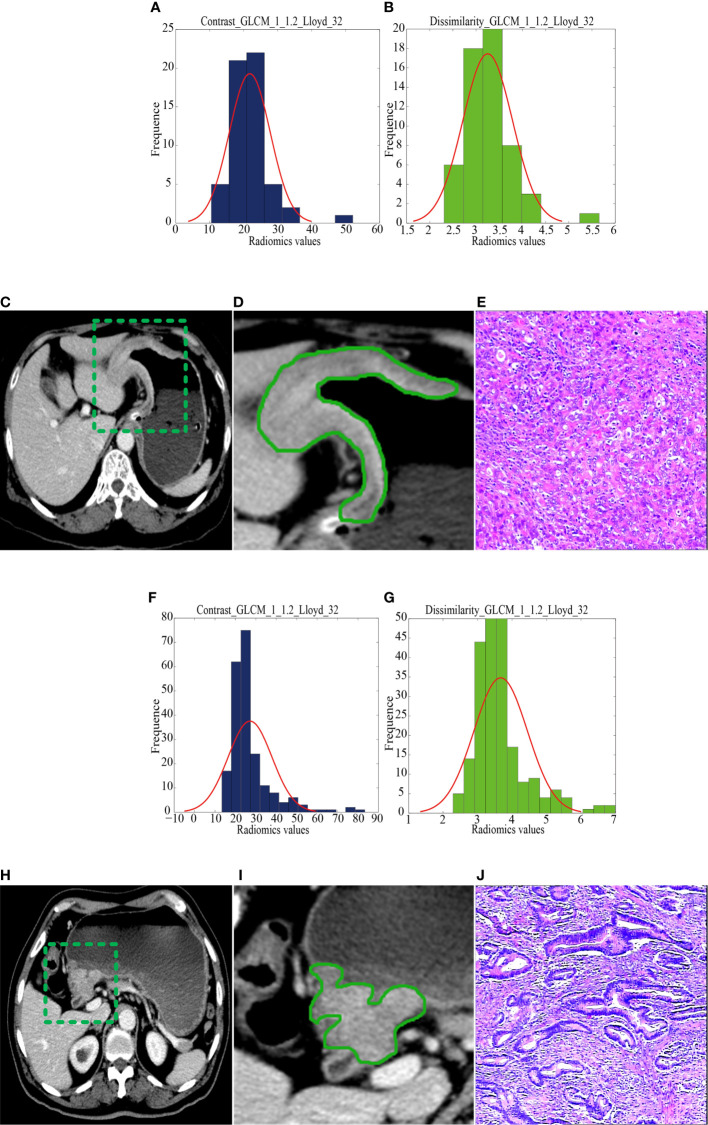
CT images and selected feature parameters in the recurrence group (RG) and nonrecurrence group (NRG). **(A)** to **(E)**, A 68-year-old man in the RG. **(A, B)**, The feature parameter maps of Contrast_GLCM_1_1.2_Lloyd_32 and Dissimilarity_GLCM_1_1.2_Lloyd_32 had average values of 21.80 ± 6.03 and 3.25 ± 0.54, respectively. **(C, D)**, The portal venous contrast-enhanced CT images showed a lesion. **(E)** This lesion was finally confirmed as diffuse-type gastric cancer by histopathological analysis (H&E, 400×). **(F)** to **(J)**, A 56-year-old man in the NRG. **(F, G)**, The feature parameter maps of Contrast_GLCM_1_1.2_Lloyd_32 and Dissimilarity_GLCM_1_1.2_Lloyd_32 had average values of 27.17 ± 10.71 and 3.67 ± 0.78, respectively. **(H, I)**, The portal venous contrast-enhanced CT images showed a lesion. **(J)** This lesion was finally confirmed as mixed-type gastric cancer by histopathological analysis (H&E, 400×).

The median radiomics scores of the RG and NRG were -0.86 (interquartile range (IQR): -1.32 to -0.32) and -1.77 (IQR: -2.21 to -1.29) in the training cohort (P < 0.001), -1.37 (IQR: -1.57 to -0.91) and -1.96 (IQR: -2.31 to -1.68) in the internal validation cohort, -1.41(-1.45,-1.40) and -1.45(-1.45,-1.44) in the external validation cohort, respectively. **Figure 2** shows the distribution of the two selected radiomics features in the RG and NRG.

### Construction of the Clinical Model and Radiomics Nomogram

**Figure 3:**
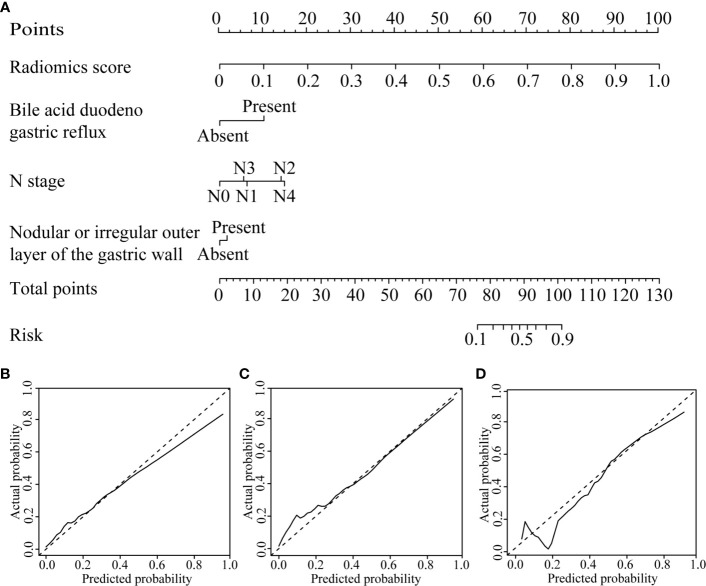
CT-based radiomics nomogram. **(A)** The radiomics nomogram was developed based on the R-score and the representative clinical risk factors. **(B–D)** Calibration curves of the nomogram in the training, internal validation and external validation cohorts, respectively.

By univariate analysis, the bile acid duodenogastric reflux, histological classification, T stage, N stage, TNM stage, Lauren classification, high enhanced serosa sign and nodular or irregular outer layer of the gastric wall were found to be clinical risk factors for the LR of GC (**Table 1**). Meanwhile, multivariate analysis found the bile acid duodenogastric reflux, T stage, N stage and nodular or irregular outer layer of the gastric wall to be independent predictors for the LR of GC in the clinical model (**Table 3**). In the combined model, which integrated the radiomics signature with the clinical risk factors, the bile acid duodenogastric reflux, N stage and nodular or irregular outer layer of the gastric wall were selected as the independent predictors (**Table 4**). Using the combined model, the radiomics nomogram was developed for the prediction of LR (**Figure 3A**).

**Table 3:** Multivariate analysis of risk factors for the clinical model.

Intercept and Variable	β	Odds Ratio (95%CI)	P value
Intercept	-6.42		<0.001
Bile acid duodenogastric reflux	1.55	4.72 (1.93–11.52)	0.001
T stage	0.61	1.83 (0.95–3.53)	0.069
N stage	0.46	1.59 (1.16 -2.17)	0.004
Nodular or irregular outer layer of the gastric wall	1.16	3.20 (1.29–7.92)	0.012

**Table 4:** Multivariate analysis of risk factors for the radiomics nomogram.

Intercept and Variable	β	Odds Ratio (95%CI)	P value
Intercept	-0.85		0.326
Bile acid duodenogastric reflux	1.64	5.14 (1.83-14.45)	0.002
N stage	0.41	1.53 (1.07 -2.14)	0.020
Nodular or irregular outer layer of the gastric wall	0.58	1.78 (0.65-4.85)	0.259
Radiomics signature	2.02	7.57 (3.35-17.09)	<0.001

### Independent Validation and Predictive Performance of the Three Models

**Figure 4:**
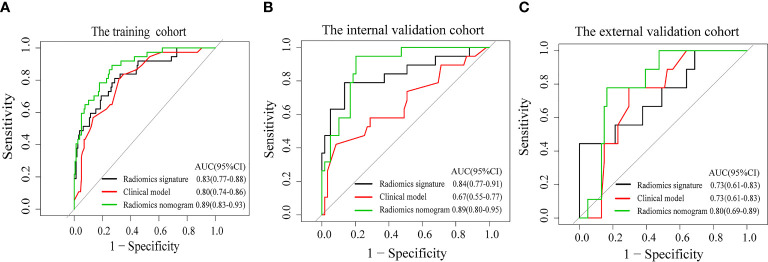
Receiver operating characteristic curves of the clinical model (red line), the radiomics signature (black line), and the radiomics nomogram (green line) in the training cohort **(A)** and the validation cohorts **(B, C)**.

The radiomics nomogram calibration curve demonstrated good agreement in three cohorts (**Figures 3B–D**). The results of ROC curve analysis of the clinical model, radiomics signature, and radiomics nomogram are summarized in **Table 5** and **Figure 4**. In the training cohort, the AUC of the clinical model was 0.80 (95% confidence interval [CI]: 0.74-0.86), and the cutoff threshold value was 0.17. The AUC of the radiomics signature was 0.83 (95% CI: 0.77–0.88), which was greater than that of the clinical model, and the cutoff threshold value was -1.41. The AUC of the radiomics nomogram was 0.89 (95% CI: 0.83-0.93), which was the highest AUC of the three models, and the cutoff value was -1.89. The AUC of the radiomics nomogram was significantly greater than both the radiomics signature and the clinical model (P < 0.05). The NRI also showed that the radiomics nomogram had a better predictive performance than the clinical model in the internal validation cohort (NRI= 0.21, P=0.003) and external validation cohort (NRI=0.40, P<0.001). According to the Hosmer–Lemeshow test, the goodness of fit was good (P > 0.05) in the all the three cohorts.

**Table 5:** Predictive performance of the clinical model, radiomics signature, and radiomics nomogram in the training and validation cohorts.

Model	Training cohort (n = 194)			Internal validation cohort (n = 78)			External validation cohort(n=70)		
	Clinical model	Radiomics signature	Radiomics nomogram	Clinical model	Radiomics signature	Radiomics nomogram	Clinical model	Radiomics signature	Radiomics nomogram
AUC	0.80	0.83	0.89	0.67	0.84	0.89	0.73	0.73	0.80
(95% CI)	(0.74-0.86)	(0.77-0.88)	(0.83-0.93)	(0.55-0.77)	(0.74-0.91)	(0.80-0.95)	(0.61-0.83)	(0.61-0.83)	(0.69-0.89)
Sensitivity	0.81	0.81	0.89	0.42	0.63	0.95	0.78	0.44	0.78
	(30/37)	(30/37)	(33/37)	(8/19)	(12/19)	(18/19)	(7/9)	(4/9)	(7/9)
Specificity	0.68	0.71	0.73	0.92	0.95	0.80	0.71	0.93	0.84
	(107/157)	(112/157)	(115/157)	(56/59)	(56/59)	(47/59)	(43/61)	(57/61)	(51/61)
Accuracy	0.71	0.73	0.76	0.79	0.87	0.83	0.71	0.87	0.83
	(137/194)	(142/194)	(148/194)	(62/78)	(68/78)	(65/78)	(50/70)	(61/70)	(58/70)
PPV	0.38	0.40	0.44	0.83	0.80	0.60	0.28	0.50	0.41
	(30/80)	(30/75)	(33/75)	(54/65)	(12/15)	(18/30)	(7/25)	(4/8)	(7/17)
NPV	0.94	0.94	0.97	0.62	0.89	0.98	0.96	0.92	0.96
	(107/114)	(112/119)	(115/119)	(8/13)	(56/63)	(47/48)	(43/45)	(57/62)	(51/53)
Delong Test	P<0.001	P<0.001	P<0.001	P<0.035	P<0.001	P<0.001	P<0.002	P<0.021	P<0.006

AUC, area under the curve; PPV, positive predictive value; NPV, negative predictive value; CI, confidence interval.

### Clinical Utility Analysis of the Prediction Models

**Figure 5:**
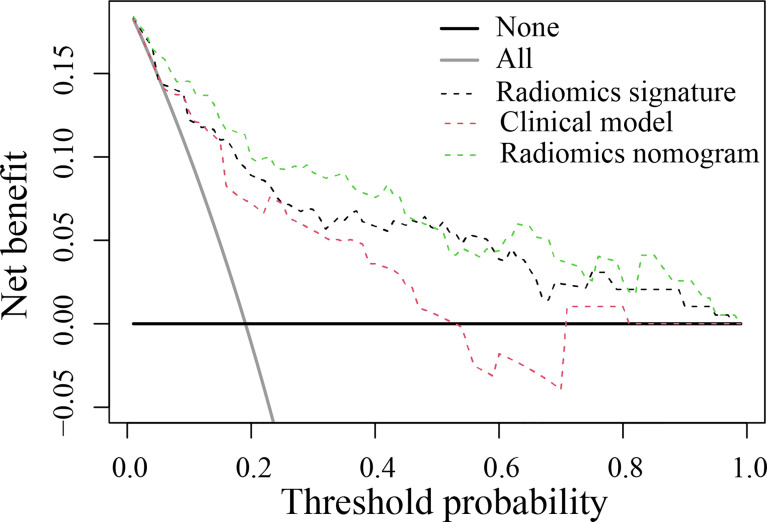
Decision curve analysis for the developed prediction models. The y-axis represents the net benefit. The x-axis represents the threshold probability. The green line represents the radiomics nomogram. The red line represents the clinical model. The black line represents the radiomics signature. The gray line represents the assumption that all patients were included in the recurrence group (RG). The black line represents the assumption that no patients were included in the RG. The threshold probability was where the expected benefit of the treatment was equal to the expected benefit of avoiding treatment. The decision curve in the validation cohort showed that the radiomics nomogram added more net benefit than the clinical model within the range of 0.01-0.98.

The DCA found that when the threshold probability was between 0.01 and 0.98, the radiomics nomogram added more net benefit than the “all patients” and “no patient” programs (**Figure 5**). Stratified analysis by the DeLong test showed that the performance (P > 0.05) of the radiomics nomogram was not affected by the age or sex of the patient, CT system, or contrast agent (**Supplementary Material Figure S2**).

## Discussion

In the present study, a novel CT-based radiomics nomogram that incorporated the radiomics signature with independent clinical risk factors (bile acid duodenogastric reflux, N stage and nodular or irregular outer layer of the gastric wall) was developed and validated, providing a better predictive performance than the clinical model and radiomics signature alone in predicting the LR of GC after radical resection. This nomogram could identify patients at a higher risk of LR based on the preoperative CT images.

Bile acids have been implicated in the development of cancer in the gastric remnant after gastrectomy (21). For example, animal research on rats by Kuwahara et al. has demonstrated that bile acids promote carcinogenesis in the residual stomach (22). Another study by Lorusso et al. has revealed that duodenogastric reflux can increase the risk of gastric stump cancer after gastric resection (23). Hence, we selected bile acid duodenogastric reflux as a clinical risk factor in this study. It was identified as an independent factor in the multivariate analysis and prediction models, suggesting that bile acid duodenogastric reflux is a robust factor in the process of LR of GC following radical resection. This finding may be due to the fact that bile acids are important toxic factors involved in injury of the gastric mucosa, playing an important role in the process of gastric carcinogenesis (24). A study by Carino et al. has shown that the bile acid receptor GPBAR1 (TGR5) is expressed in human GC and promotes epithelial–mesenchymal transition in GC cell lines (25). It also has been demonstrated that bile acids in gastric juice contribute to the progression of histologic atrophy and intestinal metaplasia without inflammatory cell infiltration, followed by carcinogenesis (26). Thus, the surveillance of bile acid duodenogastric reflux plays an important role in predicting the LR of GC among patients after radical resection.

CT is widely used in the preoperative evaluation of GC. In this study, we also analyzed the relationship between the CT signs of the lesion and LR. The results showed that the difference of high enhanced serosa sign and nodular or irregular outer layer of the gastric wall between the RG and the NRG was statistically significant. In addition, nodular or irregular outer layer of the gastric wall was selected as an independent predictor while constructing the predictive model. This may be due to the fact that these two signs are related to serosal invasion, and the lesions with these two signs may be stage T4a (27). The higher T staging, the greater probability of LR.

At present, the TNM staging system is the most commonly used tool for clinical treatment planning of cancers and predicting the prognosis of patients. Previous studies have shown that the higher T stage are important risk factors for the prediction of recurrence and survival of GC after radical resection (28). In addition, Liu et al. have reported that lymph node metastasis status is an independent risk factor for prognosis of GC after curative resection (29). Our study also demonstrated that the T stage and N stage were independent factors in developing the clinical risk factor-based prediction model. However, it is generally accepted that the TNM staging system does not take into account the heterogeneity of the tumor, which can affect the prognosis of patients (13).

Intratumor heterogeneity has been found to be related to the prognosis of patients, and precision medicine requires a better understanding of the intratumoral heterogeneity (30). In contrast to the TNM staging system and other clinical factor-based models, radiomics can extract sub-visual yet quantitative features from medical images, which can reflect the biological information such as cell morphology, gene and molecular expression, and tumor heterogeneity (9, 31). Thus, in this study, a radiomics nomogram integrating the radiomics signature with independent clinical factors (bile acid duodenogastric reflux, N stage and nodular or irregular outer layer of the gastric wall) was developed for the prediction of LR of GC. In addition, we found that the radiomics nomogram exhibited a significantly better predictive performance than the radiomics signature and clinical model alone, thereby strengthening the ability to predict the LR of GC after radical resection.

In this study, contrast_GLCM_1_1.2_Lloyd_32 and dissimilarity_GLCM_1_1.2_Lloyd_32 were selected as valuable predictors to build the radiomics signature. These two radiomics features are high order features, whose measurements are limited on low gray level intensity of voxels. Contrast_GLCM_1_1.2_Lloyd_32 is a measure of the local intensity variation with a larger value correlates with a greater disparity in intensity values among neighboring voxels. Dissimilarity_GLCM_1_1.2_Lloyd_32 is a measure of local intensity variation defined as the mean absolute difference between the neighbouring pairs. A larger value correlates with a greater disparity in intensity values among neighboring voxels. Interestingly, in the current study, the NRG group had larger values in contrast_GLCM_1_1.2_Lloyd_32 and dissimilarity_GLCM_1_1.2_Lloyd_32 features than the RG group, which may be attributed to the necessity of various low gray-level signal intensities to represent the low-risk tissues contained in the NRG group.

The present study has several limitations that must be acknowledged. First, this was a retrospective study, and selection bias may not have been completely avoided. So, the performance of the prediction model may be magnified. Second, manual segmentation of the tumor was challenging and contentious. Introducing semi-automated or automated segmentation methods may improve the reproducibility of segmentation. Third, although the AUC of the radiomics nomogram was the highest, the sensitivity and specificity of this nomogram were still below 90%.

In conclusion, bile acid duodenogastric reflux and tumor heterogeneity are important risk factors in predicting the LR of GC. The CT-based radiomics nomogram, which integrated the CT-based radiomics signature and independent clinical risk factors, could be used as a potential biomarker for the individualized prediction of LR of GC after radical resection. Future multicenter prospective studies are required to validate the findings of this study.

## Publisher’s Note

All claims expressed in this article are solely those of the authors and do not necessarily represent those of their affiliated organizations, or those of the publisher, the editors and the reviewers. Any product that may be evaluated in this article, or claim that may be made by its manufacturer, is not guaranteed or endorsed by the publisher.

## Data Availability Statement

The original contributions presented in the study are included in the article/**Supplementary Material**. Further inquiries can be directed to the corresponding authors.

## Ethics Statement

The studies involving human participants were reviewed and approved by the institutional review board of Jiangmen central hospital and the First Affiliated Hospital of Sun Yat-sen University. The ethics committee waived the requirement of written informed consent for participation.

## Author Contributions

LH, WL, and YLi designed the research. LH, QC, WH, HX, TZ, and XL collected the data. BF, YLiu, YC, and CL contributed data analysis tools and performed the analysis, BF also acquired the funding. LH and YLi wrote the paper. WL and S-TF supervised the study. All authors contributed to the article and approved the submitted version.

## Funding

This work was supported by the National Natural Science Foundation of China [grant numbers: 81960324 (to Bao Feng)] and the incubation project of 1000 Young and Middle-aged Key Teachers in Guangxi Universities [grant numbers: 2018GXQGFB160 (to Bao Feng)], and the outstanding scientific research youth project of Jiangmen Central Hospital [grant numbers: J202005 (to Liebin Huang)].

## Conflict of Interest

The authors declare that the research was conducted in the absence of any commercial or financial relationships that could be construed as a potential conflict of interest.

## Publisher’s Note

All claims expressed in this article are solely those of the authors and do not necessarily represent those of their affiliated organizations, or those of the publisher, the editors and the reviewers. Any product that may be evaluated in this article, or claim that may be made by its manufacturer, is not guaranteed or endorsed by the publisher.

## Abbreviations

GC, gastric cancer; AUC, area under the curve; ROC, receiver operating characteristic; DCA, decision curve analysis; ROI, region of interest; ICCs, intraclass correlation coefficients; LASSO, least absolute shrinkage and selection operator; R-score, radiomics score; PPV, positive predictive value; NPV, negative predictive value; NRI, net reclassification index; RG, recurrence group; NRG, nonrecurrence group; GLCM, gray level co-occurrence matrix.
