# Supplementary material for: Computed Tomography-Based Radiomics Nomogram: Potential to Predict Local Recurrence of Gastric Cancer After Radical Resection
Source: Front Oncol. 2021 Sep 2;11:638362. doi: 10.3389/fonc.2021.638362 (PMC8445075; doi:10.3389/fonc.2021.638362)
Supplement: Supplementary file 1 [file DataSheet_1.doc]

**Supplementary** **material**

**Figure S1. Enrollment procedure**

Other factors

Center 1

2008.10~2017.07, n =656

Center 2

2015.03 ~2017.04, n=158

95 and 29 patients were excluded from center 1 and center 2 due to CT factors, respectively. ①contrast-enhanced abdominal CT longer than two weeks before operation, n=26 vs. 16

②unsatisfactory gastric distention, n=30 vs. 9

③insufficient quality of CT imaging, n=39 vs. 4

Further review for eligibility, n=561

Further review for eligibility, n=129

CT factors

CT factors

130 and 21 patients were excluded from center 1 and center 2 due to Operation factors, respectively.

①the surgery didn’t meet the standard of D2 lymphadenectomy and R0 resection, n=69 vs. 13

②less than 15 retrieved lymph nodes, n=61 vs. 8

Operation factors

Operation factors

Patients with GC who had received surgery and preoperative abdominal contrast-enhanced CT examination

Further review for eligibility, n=431

Further review for eligibility, n=108

Other factors

159 and 38 patients were excluded from center 1 and center 2 due to Operation factors, respectively.

①incomplete medical records, n=48 vs. 9

②had previous treatment with chemotherapy or radiotherapy before surgery, n=66 vs. 10

③follow-up shorter than 2 years before LR, n=45 vs. 19

Finally recruited, n=272

Finally recruited, n=70

**Table S1**. **Radiomics score (Rad-score) formula**

Rad-score=-1.41426-0.000900*Contrast_GLCM_1_1.2_Lloyd_32

-0.00212346*Dissimilarity_GLCM_1_1.2_Lloyd_32

| **Group** | **Feature** | **Formula** |
| --- | --- | --- |
| **GLCM** | Contrast |  |
|  | Dissimilarity |  |

Notes:

GLCM: Gray-level co-occurrence matrix.

A: Contrast

B: Dissimilarity

is the number of discrete intensity levels in the image. represents the number of times that a voxel with gray level and gray level are adjacent to each other in the image.

**A1. The process of feature selection based on the least absolute shrinkage and selection operator (LASSO)**

LASSO is a linear regression method, which realizes the feature selection by introducing 1 norm. In the LASSO, each feature corresponds to a weighting coefficient, which represents the importance of the feature.

A full regression model for k independent variables with weighting coefficients β is:

(1)

Where the outcome Y is either the original dependent variable Y, which is the predictive value of model.

The sum of absolute values of the weighting coefficients is then estimated with the following restriction:

(2)

where $\lambda$ is the “tuning” parameter. As λ approaches indefinite, it has no effect and the solutions are estimates for the full and unrestricted model. For smaller $\lambda$ values, solutions are shrunken versions of the estimates, with many weighting coefficients decreased to the 0 value. The selected value of $\lambda$ was calculated by using cross-validation. When the optimal the “tuning” parameter (i.e. the value of λ minimizing cross-validation error) is $\lambda_{best}$, the corresponding features are selected, which are the valuable predictors.

The process of the feature selection consists of three steps: First, based on the LASSO method, the weighting coefficients corresponding to each feature were calculated, and the corresponding features were selected according to the distribution of non-zero weighting coefficients. Secondly, the adjustment parameters $\lambda$ were selected by the generalized cross validation method. Finaly, when the optimal adjustment parameter is$\lambda_{best}$, the corresponding features are selected, which are the valuable predictors.

**A2. Stratified analysis**

In order to evaluate robustness of the radiomics nomogram, we performed stratified analysis based on the gender, age, CT system and contrast agent. We used the ROC curve and AUC to assess the performance of radiomics nomogram. The results showed that radiomics nomogram was not influenced by these factors.

1. Stratified analysis of the age

The AUCs of the age (< 60 years and ≥ 60 years) were 0.8847 and 0.8475 **(Figure S2. A)**, respectively. The p values of these two groups compared with the overall cohorts were 0.6987 and 0.6594 by Delong test.

1. Stratified analysis of the gender

The AUCs of the gender (Male and Female) were 0.8688 and 0.8722 **(Figure S2. B)**, respectively. The p values of these two groups compared with the overall cohorts were 0.9933 and 0.9436 by Delong test.

1. Stratified analysis of the CT system

The AUCs of the CT system (Aquilion one-64 and Somatom force) were 0.8565 and 0.8823 **(Figure S2. C)**, respectively. The p values of these two groups compared with the overall cohorts were 0.7767 and 0.7616 by Delong test.

1. Stratified analysis of contrast agent

The AUCs of the contrast agent (3.0 mL/s and 3.5 mL/s) were 0.8841 and 0.8568 **(Figure S2. D)**, respectively. The p values of these two groups compared with the overall cohorts were 0.7203 and 0.7878 by Delong test.

**Figure S2. The ROCs with radiomics nomogram score for each group stratified by the age (A), sex (B), CT system (C) and contrast agent (D).**

**
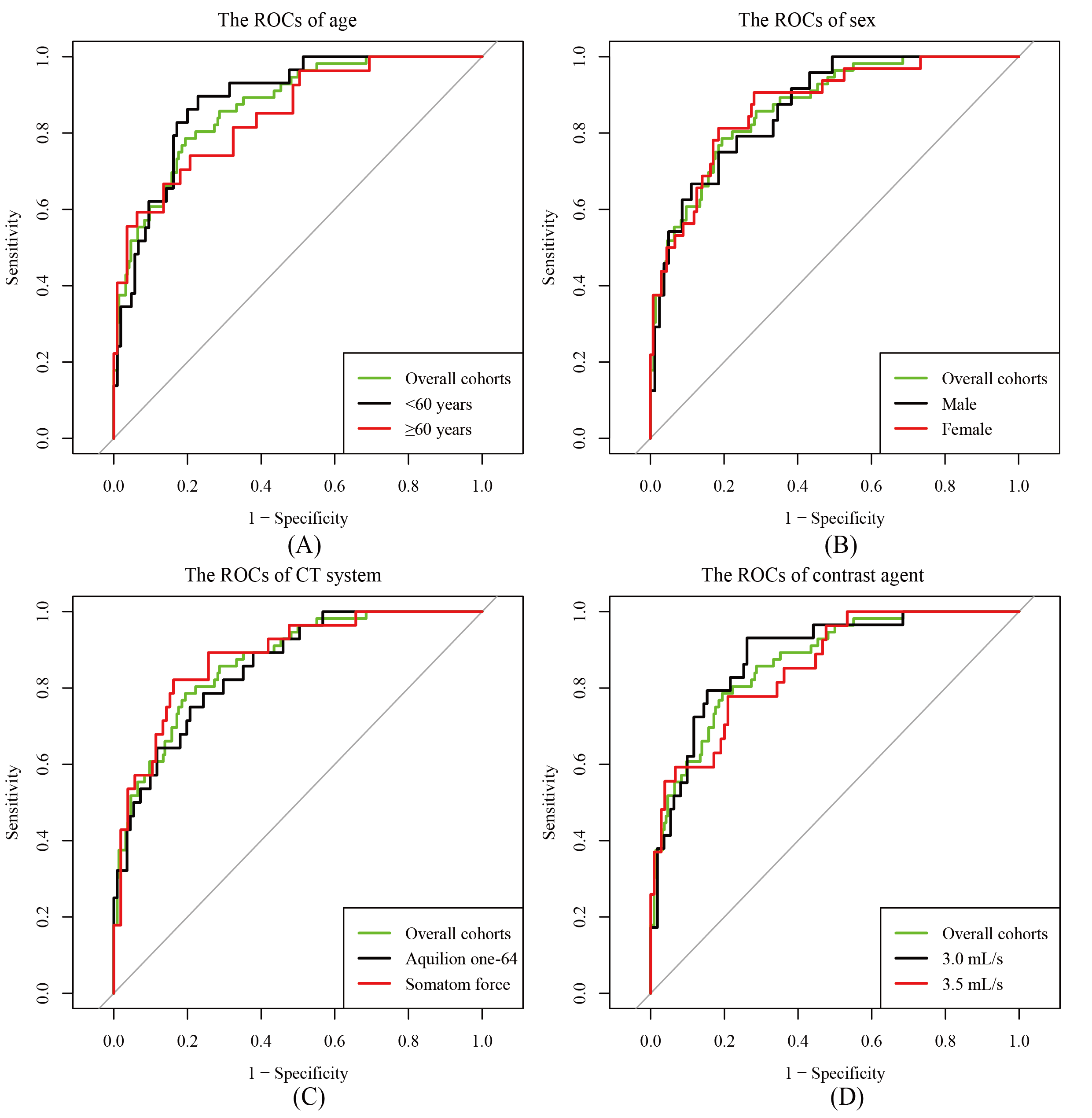
**
